# Intermittent fasting activates macrophage migration inhibitory factor and alleviates high-fat diet-induced nonalcoholic fatty liver disease

**DOI:** 10.1038/s41598-023-40373-5

**Published:** 2023-08-11

**Authors:** Dezhao Li, Yaoshan Dun, Dake Qi, Jeffrey W. Ripley-Gonzalez, Jie Dong, Nanjiang Zhou, Ling Qiu, Jie Zhang, Tanghao Zeng, Baiyang You, Suixin Liu

**Affiliations:** 1 Division of Cardiac Rehabilitation, Department of Physical Medicine and Rehabilitation, Xiangya Hospital Central South University, Changsha, Hunan China; 2 National Clinical Research Center for Geriatric Disorders, Xiangya Hospital Central South University, Changsha, Hunan China; 3 College of Pharmacy, University of Manitoba, Winnipeg, MB Canada

**Keywords:** Obesity, Obesity

## Abstract

Switching to normal diet (ND) is the regular therapy for high-fat diet (HFD)-induced nonalcoholic fatty liver disease (NAFLD). Intermittent fasting (IF) is a unique treatment which may exhibits better therapeutic efficacy. Thus, we aim to investigate the therapeutic effects of these treatments and exploring the mechanisms. In the present study, NAFLD mouse model was induced by a 10-week HFD. Thereafter, mice adopted continued HFD, ND, or IF for the next 12 weeks. Finally, the liver was then harvested to assess lipid deposition, lipid metabolism, apoptosis, and autophagy, while blood was collected to determine blood glucose and insulin. The results showed that IF and ND treatment improved lipid deposition and metabolic disorder of NAFLD mice; the increasing body weight, liver weight, and HOMA-IR index of HFD mice were also alleviated by IF and ND. Furthermore, IF and ND treatment activated the macrophage migration inhibitory factor (MIF)/AMPK pathway and regulated its downstream autophagy and apoptosis. However, the efficacy of IF was better than ND. Both IF and ND activates MIF signaling and alleviate the lipotoxicity of NAFLD while IF therapy is more effective than ND. The different MIF up-regulation might be the underlying mechanism of why IF benefits more than ND.

## Introduction

Rising caloric intake and sedentary lifestyles have contributed to the increased prevalence of metabolic disorders, including Non-alcoholic fatty liver disease (NAFLD), which is marked by excessive lipid accumulation in hepatocytes and not only leads to liver inflammation and fibrosis^1^ but also is closely related to insulin resistance, type 2 diabetes, obesity and hyperlipidemia^2^, which greatly increases the risk of cardiovascular disease and is an urgent clinical problem. Normal diet (ND) therapy is the primary treatment for high-fat diet (HFD)-induced complications of NAFLD; however, simply adopting an ND after prolonged HFD exposure may not fully reverse these changes^3,4^, and the mechanism remains unknown. Intermittent fasting (IF), a dietary strategy alternating between fasting and eating, has gained attention for its health benefits, especially in managing metabolic disorders among overweight/obese patients^2^. Several studies have highlighted IF's therapeutic potential in NAFLD^5,6^, attributing its efficacy to metabolism regulation^7^. Nevertheless, a direct comparison of IF and ND treatments for NAFLD is needed, along with further exploration of underlying mechanisms.

Hepatocyte apoptosis is a critical event in NAFLD pathophysiology, as apoptotic hepatocytes stimulate immune cells and hepatic stellate cells to produce proinflammatory cytokines, leading to metabolic abnormalities and fibrosis^8^. Macrophage migration inhibitory factor (MIF), an immunoregulatory cytokine, plays a role in modulating inflammation response and metabolism^9^. MIF has been shown to promote AMP-activated protein kinase (AMPK) phosphorylation and reduce liver fibrosis in a CD74-dependent manner^10,11^. Additionally, MIF has been found to prevent apoptosis in cardiomyocytes by regulating the MKK4/JNK pathway^12^, and our previous research confirmed that endogenous hepatic MIF prevents hepatocyte apoptosis in NAFLD through modulation of the same pathway^13^. However, it remains unclear whether this mechanism underlies the benefits of ND and IF.

AMPK, a downstream pathway of MIF, facilitates protective cellular autophagy^14,15^. Studies have identified AMPK-dependent lipophagy as a therapeutic target for NAFLD in exercise treatment^16^, and MIF/AMPK signalling activation has been shown as a mechanism by which exercise prevents NAFLD^17^. Given the similar energy metabolism regulation between dietary restriction and exercise, we hypothesise that MIF/AMPK-mediated autophagy may also be one of the mechanisms by which diet treatment counteracts hepatocyte apoptosis in NAFLD.

In light of these observations, this study aims to compare ND and IF treatments for NAFLD and investigate their potential to combat hepatocyte apoptosis via MIF/AMPK signaling. We hypothesize that autophagy and apoptosis regulation underlie the therapeutic effects in NAFLD.

## Results

### Dietary interventions relieve liver lipid accumulation induced by HFD

**Figure 1:**
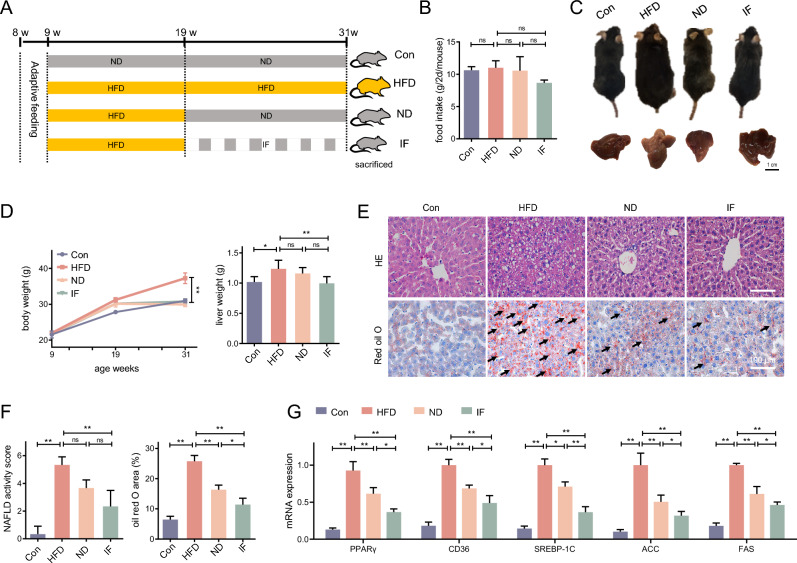
Dietary interventions relieve liver lipid accumulation induced by HFD. (**A**) Following 1-week adaptive feeding, mice were fed a high-fat diet (HFD) for 10 weeks, and shifted o another 12-week HFD, ND, or intermittent fasting (IF); (**B**,**C**) Pictures of mice in different groups were taken before sacrifice, and the body weight was monitored and recorded; The livers pictures were taken and the weight of them were recorded; (**D**,**E**) H&E staining and oil red O staining of the liver tissue slices were performed, and the oil red O positive area (%) of each view was indicated by arrows, the NAFLD activity score and oil red O area of each field was analyzed; (**F**) The mRNA expression of PPARγ, CD36, SREBP-1C, ACC, and FAS in the liver were determined. Data are expressed as mean ± SD, n = 3–6. *, ** represent *P* < 0.05, *P* < 0.01.

Following the adaptive feeding, mice were fed HFD for 10 weeks, then their dietary mode was shifted to another 12-week HFD, ND, or IF (Fig. 1A). Following dietary switching and throughout the intervention period, food intake was weighed in each group for 2 days as a fasting cycle. Food intake in IF exhibited decreasing trend, but statistical significance was not observed (Fig. 1B). It was found that the body weight and liver weight were increased in HFD mice as expected, while these alterations induced by HFD were prevented by dietary substitution to ND or IF (Fig. 1C,D). In the HE staining image of liver slices, hepatocellular ballooning and slight lobular inflammation were observed in HFD, ND and IF liver slices (Fig. 1E,F). Moreover, multiple transparent cavities were observed in the HFD mice, which proved to be lipid droplets stained by oil red O. The NAFLD activity score and oil red O area of each group was assessed, the results showed an activity score greater than 5 in the HFD group, which is consistent with NASH, while the score of IF was much lower. However, a statistically significant difference between ND with HFD or IF was not observed. Furthermore, compared to HFD the lipid droplets were fewer and smaller in size in the ND and IF groups, and the oil red O area in the IF liver were smaller than in ND liver. Interestingly, the therapeutic effect of an additional 12-week IF on liver lipid deposition was better than ND. Moreover, it shows a similar trend in the mRNA expressions of lipogenesis-related genes, PPARγ, CD36, SREBP-1C, ACC, and FAS. Dietary interventions decreased HFD-elevated lipogenesis, while IF treatment exhibited a better efficacy (Fig. 1G).

### Dietary interventions relieve liver metabolic disorder in HFD mice

**Figure 2:**
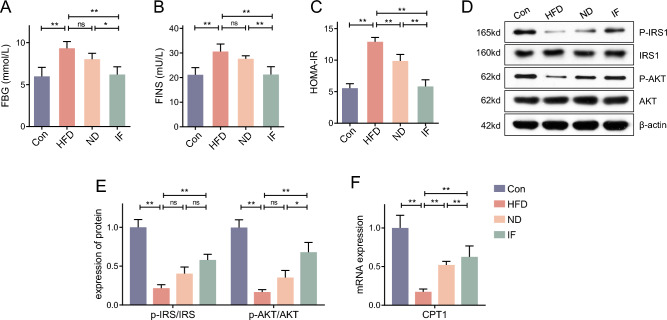
Dietary interventions relieve liver metabolic disorder in HFD mice. (**A**,**B**) Following the 22-week intervention, the blood of mice was collected after 12-h fasting, then the fasting blood glucose (FBG) and the fasting blood insulin (FINS) were assessed; (**C**) The HOMA-IR index was calculated by the formula; (**D**) The protein expression of p-IRS, IRS, p-AKT, AKT in mice liver were determined; (**E**) The mRNA expression of CPT1 was determined. Data are expressed as mean ± SD, n = 3–6. *, ** represent *P* < 0.05, *P* < 0.01.

We further assessed the insulin resistance and lipid metabolic process of these mice. As shown in Fig. 2A–C, the HOMA-IR index was increased in HFD mice when compared with Con mice, while it was lower in ND and IF mice comparing with HFD mice. We found that phosphorylation of IRS and AKT were inhibited in HFD mice, and an additional 12-week ND or IF could rescue the inhibition (Fig. 2D,E). In keeping with the result of Fig. 1E, the IF intervention exhibits a greater anti-insulin resistance ability. As for the lipid metabolic disorder, we assessed the fatty acid oxidation related gene CPT1 (Fig. 2F). In HFD mice, there was more fatty acid oxidation inhibition when compared to normal mice. These metabolic abnormalities could be prevented by 12-week ND or IF, and the IF mice exhibited healthier lipid metabolism than ND mice.

### MIF/AMPK-mediated autophagy in the liver was activated by intermittent fasting

**Figure 3:**
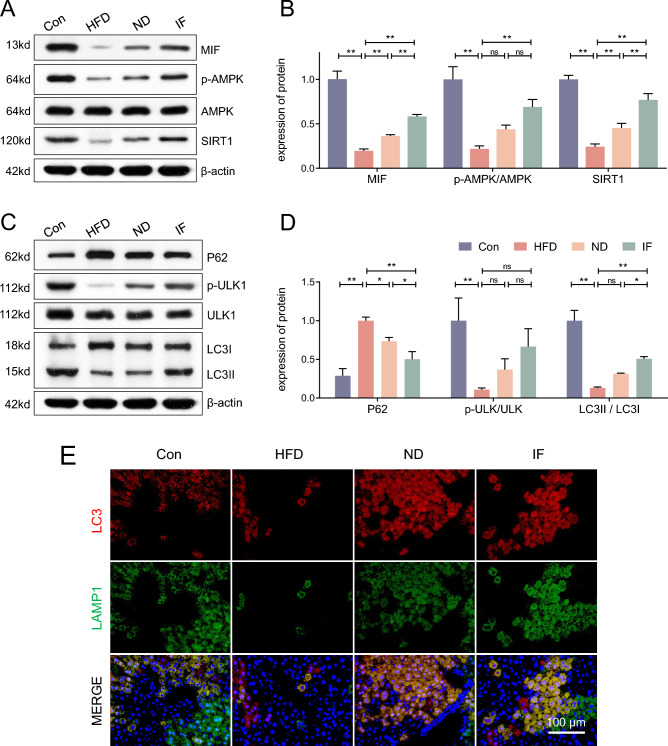
MIF/AMPK-mediated autophagy in the liver was activated by intermittent fasting. (**A**,**B**) Following the 22-week intervention, the protein expression of MIF, p-AMPK, AMPK, and SIRT1 in the liver were determined and analyzed; (**C**,**D**) The protein expression of P62, p-ULK1, ULK, LC3I, and LC3II in the liver were determined and analyzed; (**E**) Representative images of LC3 and LAMP1 were taken, scale bar = 100 μm. Data are expressed as mean ± SD, n = 3. *, ** represent P < 0.05, P < 0.01.

As shown in Fig. 3A, protein expression of MIF was decreased in HFD mice and rescued following a 12-week ND and IF intervention. Moreover, the downstream AMPK and SIRT1 were also determined. Our results show that the AMPK/SIRT1 pathway was inhibited in HFD mice while 12 weeks of ND and IF reversed this change, with the IF intervention showing greater effectiveness (Fig. 3B).

Autophagy was further assessed in the mice. We assessed the protein expression of key autophagy markers P62, p-ULK1/ULK1, LC3II/LC3I (Fig. 3C,D). Then, the representative immunofluorescence images of LC3 and LAMP1 were taken (Fig. 3E). These results showed that autophagy was inhibited in HFD mice when compared to the Con mice, while the autophagy level of ND mice picked up. However, the 12-week IF intervention improved the level of autophagy most, which may be the potential therapeutic mechanism.

### Hepatocyte apoptosis was relieved by intermittent fasting

**Figure 4:**
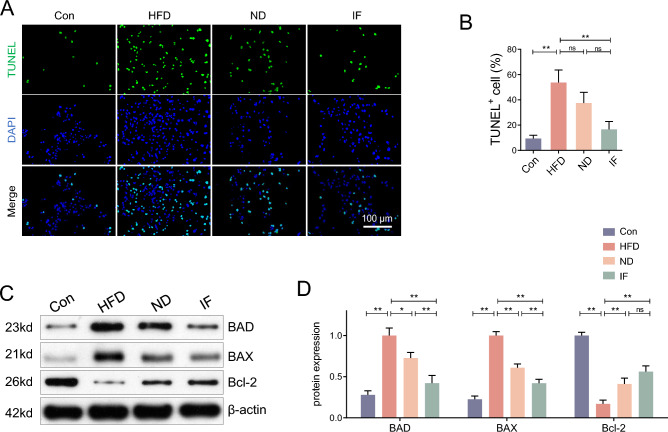
Hepatocyte apoptosis was relieved by intermittent fasting. (**A**) Following the 22-week intervention, TUNEL staining was performed in liver slices, scale bar = 100 μm; (**B**) The TUNEL positive cell numbers were analyzed; (**C**,**D**) The protein expression of BAD, BAX, and Bcl-2 in the liver were determined and analyzed; Data are expressed as mean ± SD, n = 3–6. *, ** represent P < 0.05, P < 0.01.

Apoptosis of hepatocytes plays a role in liver metabolism and NAFLD. TUNEL staining (Fig. 4A,B) and apoptosis marker, BAD, BAX, and Bcl-2 (Fig. 4C,D) were determined to assess hepatocyte apoptosis. The results showed that compared to a normal diet, HFD promoted apoptosis in the liver, However, a 12-week ND relieved this abnormality, and the IF treatment provided the greatest relief.

## Discussion

Our study's primary findings reveal that both IF and ND treatments alleviate NAFLD, with IF exhibiting a stronger therapeutic effect. This superior efficacy is attributed to IF's ability to activate MIF signaling, which in turn promotes AMPK-mediated autophagy and reverses HFD-induced apoptosis in the livers of NAFLD mice. Furthermore, IF treatment mitigates metabolic disorders in the livers of HFD mice by activating MIF signaling. These results collectively underscore the crucial role of MIF signaling and its downstream autophagy/apoptosis pathways in IF's therapeutic impact on NAFLD. The HFD NAFLD model in mice simulated a phenotype similar to the human condition, characterized by obesity, insulin resistance, and hyperlipidemia^18^. Excessive intake of FFA directly resulted in triglyceride accumulation in the liver^19^. HFD with 45% calories coming from fat (mainly lard) was used in this study, which induced obesity, insulin resistance, and lipid deposition in the liver as expected. Apoptosis, programmed cell death, and autophagy, the protective repairment process form a self-regulating system against stress. The present study found increased apoptosis and impaired autophagy were in the NAFLD model, consistent with previous studies^13,20^.

The Dietary Guidelines for Americans recommend that energy requirements should be met primarily through nutrient-rich foods and beverages, with limited consumption of sugars, saturated fats, and sodium^21^. Numerous studies have confirmed the therapeutic effects of adopting a ND on NAFLD, with mechanisms linked to AMPK/ULK1 pathway-mediated lipophagy and upregulation of lipolytic factors^16,22^. In our study, a 12-week ND treatment reversed about 50% of liver lipid accumulation caused by HFD, while IF showed comparatively greater effects with 74% reversed. Moreover, some HFD-induced complications, such as impaired sperm quality, were not improved by ND treatment^3^. Our study also found that while HFD induced IRS1/AKT signaling inhibition, IF alleviated this inhibition, but no statistical difference was observed between ND and HFD mice. Consequently, IF emerges as an alternative dietary strategy that may offer greater benefits for NAFLD patients.

Various forms of IF including time-restricted feeding, alternate-day fasting, 5:2 fasting, and fasting-mimicking diet have been proved effective on NAFLD^23^. Further research is needed to compare the effectiveness of different forms of IF to provide more robust evidence for clinical applications. Clinical studies in healthy populations have shown that alternate day fasting exhibits adequate safety and provides extensive benefits on metabolic markers up to 6 months post intervention^24^. Further trials have demonstrated the efficacy of this approach in NAFLD patients, where significant reductions in hepatic steatosis and fibrosis have been noted^25^. Another trial found alternate-day IF in NAFLD patients could relieve hepatic steatosis, while lean mass, aspartate transaminase, HbA1c, plasma lipids, and hepatokines did not differ between groups, which also confirmed the safety of IF^26^. These human studies have been performed to investigate the potential benefits and risks of intermittent fasting for liver diseases, animal studies however, can provide valuable insights into underlying mechanisms. In this research, we adopted alternate-day fasting, wherein fasting is undertaken every other day. Similar to previous research, in the process of IF, food intake through each 2-day fasting cycle was comparable to the overall intake of 2-day ND^27,28^. While the body weight of ND and IF mice had shown no significant difference, IF mice exhibited reduced lipid deposition and metabolic disorder compared to ND mice, which suggests that IF as a treatment in NAFLD may be directly treating the disease itself rather than through the reduction of total energy intake.

MIF, an apoptosis-inhibiting factor, plays a significant role in various organs and cells^29,30^. MIF could be synthesized and even released from several tissues under hypoxic or ischemic conditions^31,32^. Exercise is one of the simulating treatments to induce the protective effects of MIF signaling and prevent NAFLD^17^. We assume that the moderate energy shortage as a result of IF is comparable to the calorie expenditure induced by exercise, which activates the MIF pathway. In the present study, long-term exposure to a HFD had led to a decrease in hepatic MIF protein expression due to energy oversupply, and the MIF expression was then subsequently upregulated following both IF and ND treatments. However, IF resulted in a greater increase in MIF expression than ND (1.6-fold change, IF vs. ND). We postulate that the downstream AMPK/SIRT1 signaling pathway could be instrumental in how MIF activates protective autophagy and inhibits apoptosis. In our study, the impaired AMPK/SIRT1 signaling, reduced autophagy levels, and hepatocyte apoptosis induced by HFD were mitigated by both IF and ND treatments. Notably, IF resulted in stronger autophagy and less apoptosis than ND, paralleling the greater increase in MIF expression. Therefore, it is suggested that the differential upregulation of MIF may be a potential mechanism explaining why IF offers more benefits than ND in treating NAFLD.

There are limitations in the present study. MIF release could be one of the beneficial mechanisms of IF. However, this study could not clarify all of the potential sources of MIF protein, which may leave out possible target organs of IF. This study did not compare the effects of dietary, exercise, and the combined treatment on NAFLD. Considering that dietary and exercise respectively related to the energy uptake and consumption, further research clarifies the similarities and differences regarding their mechanisms of action will help developing the best combination therapy.

To conclude, both ND and IF effectively reduce liver lipid deposition and metabolic disorder of HFD-induced NAFLD However, demonstrates superior outcomes, potentially due to its heightened activation of MIF-regulated autophagy and apoptosis in hepatocytes, presenting a potential therapeutic target for NAFLD. Nevertheless, considering high acceptance and compliance of ND treatment against the efficacy of IF therapy, this research provides evidence for individualized dietary treatment to optimize therapeutic outcomes in NAFLD.

## Materials and methods

### Ethics statement and animal experimentation

Twenty-four C57BL/6J male mice (8 weeks old) were purchased from the Department of Animals Laboratory of Central South University (Changsha, China). A 22 °C ± 2 °C temperature-controlled and 12:12-h light–dark cycle room housed the mice with free access to drinking water and food.

Mice were randomly divided into four groups after the 1-week adaptive feeding, (n = 6 each): (i) normal control (Con); (ii) high-fat diet (HFD); (iii) normal diet (ND); (iv) intermittent fasting (IF) of normal diet. Some mice were fed ND (18% calories from fat) as control, while some fed HFD (45% calories from fat) for 10 weeks to induce NAFLD. Following that, the NAFLD mice adopted HFD, ND, or IF (fasting: eating, 24 h: 24 h) for the next 12 weeks. All food was removed on the fasting days of the IF mice, while normal diet food supply was unlimited on alternate days. The detailed protocol is summarized in Fig. 1A.

After the 23-week intervention, mice were euthanized via 1% pentobarbital sodium (150 mg/kg, i.p.) after 12-h fasting. Liver and blood were collected for experiments.

All the procedures involving mice were conducted under the guidelines for the use of live animals of the National Institute of Health, and any unnecessary animal suffering was avoided. The protocols were approved by the Experimental Animal Welfare Committee of Central South University (SYXK 2020-0019). Furthermore, this study was conducted according with the ARRIVE guidelines, and all methods were performed in accordance with the relevant guidelines and regulations.

### HE staining and oil red O staining

As previously described^33^, a portion of the liver tissue was fixed, paraffin-embedded, and sliced. Then, tissue slices were stained with hematoxylin and eosin (H&E). For the oil red O staining, slices were stained by oil red O dyestuff (Wellbio, Changsha, China).

### NAFLD activity score

NAFLD activity score is a system which measures changes to NAFLD according to several histologic structure characteristics, and is scored as follows: steatosis (0–3), lobular inflammation (0–3), hepatocellular ballooning (0–2)^34^.

### HOMA-IR index

As previously performed^35^, fasting blood glucose and blood insulin were detected by glucometer (Roche Diagnostics, Indianapolis, USA) and ELISA (CSB-E05071m, Wuhan, China). The formula used to calculate the HOMA-IR index was: fasting glucose (mmol/L) × fasting insulin (mU/L)/22.5^36^.

### RNA preparation and quantitative RT-PCR

The extraction kit (Trizol, Thermo, USA) was used to extract RNA from tissue. Then, a real-time reverse transcription (RT-PCR) kit (Cwbiotech, Beijing, China) was used to convert RNA into cDNA. The primers are listed in Table 1. β-actin was regarded as a reference gene.

**Table 1:** Sequence of primers.

Genes	Species	Sequence
FAS	Mouse	F-ACCGCCATCTATATCGACCCT R-ACCACCAGAGACCGTTATGCC
PPARγ	Mouse	F-ACCTGAAGCTCCAAGAATACCAA R-ATGCTTTATCCCCACAGACTCG
CD36	Mouse	F-AAGTTGCCATAATTGAGTCCT R-CTTTAAGGTCGATTTCAGATCCG
SREBP-1c	Mouse	F-GGCCTGACAGGTGAAATCGG R-CTCAGGAGAGTTGGCACCTG
CPT1A	Mouse	F-CTTCAATACTTCCCGCATCCCT R-AGCAGCCTCCCGTCATGGTA
ACC	Mouse	F-ATGCTATTTCTTTGTTTGGTCGT R-CCCAGCACTCACATAACCAAC
β-actin	Mouse	F-ACATCCGTAAAGACCTCTATGCC R-TACTCCTGCTTGCTGATCCAC

### Protein preparation and Western blotting

As previously described^33^, the protein was extracted by grinding machine (KZ-II, Servicebio, Wuhan, CN) and radioimmunoprecipitation assay (RIPA) buffer containing protease inhibitor (Beyotime, Shanghai, China). The protein concentration was measured by the BCA Protein Assay kit (Beyotime).

After SDS-PAGE, the proteins were transferred to PVDF membranes (0.45 μm pores, Millipore, MA, USA). After blocking, the membranes were incubated with primary antibodies against p-IRS (1:500, Proteintech, USA), IRS (1:1000, Proteintech), p-AKT (1:2000, Proteintech), AKT (1:2000, proteintech), MIF (1:1000, Abcam, Cambridge, UK), p-AMPK (1:1000, Abcam), AMPK (1:1000, Abcam), SIRT1 (1:500, Proteintech), P62 (1:1000, Proteintech), p-ULK1 (1 µg/ml, Abcam), ULK1 (1 µg/ml, Abcam), LC3 (1:1000, Proteintech), BAD (1:1000, Abcam), BAX (1:1000, Abcam), Bcl-2 (1:1000, proteintech, USA), β-actin (1:5000, Proteintech) respectively. Following incubation of HRP-labeled secondary antibody (1:5000, Proteintech), the band pictures were collected and analyzed by the gel documentation system (Bio-Rad, CA, USA).

### TUNEL

The terminal deoxynucleotidyl transferase-mediated 2′-deoxyuridine 5′-triphosphate nick-end labeling (TUNEL) assay staining was performed following the manufacturer’s instructions. The liver sections were prepared and stained with reagents of a TUNEL kit (Yeasen Biotechnology, Shanghai, China). TUNEL positive cells portion was analyzed.

### Immunofluorescent staining

After being dewaxed and hydrated, liver sections were blocked in 5% bovine serum albumin (BSA) with 0.1% Triton X-100 (SigmaAldrich). After rinsing in PBS, they were incubated with primary antibodies against LAMP1 (1:50, CST), and LC3 (1:50, Abcam). After rinsing again, the secondary antibodies (1:400, Proteintech) were incubated in the dark. Finally, staining DAPI (SigmaAldrich) and images were acquired using the fluorescence microscope (Eclipse, Nikon, JPN).

### Statistical analysis

Statistical analyses were performed with Prism 8 software (GraphPad, Inc., CA, USA). The results are expressed as mean ± SD. One-way ANOVA plus the Bonferroni test were used for analyses. *P* < 0.05 represents statistical significance.

### Supplementary Information

Supplementary Information.

## Data Availability

The datasets for this study can be found in the supplementary material.

## References

[1] Mantovani A, Dalbeni A (2021). Treatments for NAFLD: State of Art. Int. J. Mol. Sci..

[2] Patikorn C (2021). Intermittent fasting and obesity-related health outcomes: An umbrella review of meta-analyses of randomized clinical trials. JAMA Netw. Open..

[3] Crisostomo L (2019). A switch from high-fat to normal diet does not restore sperm quality but prevents metabolic syndrome. Reproduction.

[4] Littlejohns B, Lin H, Angelini GD, Halestrap AP, Suleiman MS (2014). Switching back to normal diet following high-fat diet feeding reduces cardiac vulnerability to ischaemia and reperfusion injury. Cell Physiol. Biochem..

[5] Rozanski G (2021). Effect of different types of intermittent fasting on biochemical and anthropometric parameters among patients with metabolic-associated fatty liver disease (MAFLD)—A systematic review. Nutrients.

[6] de Cabo R, Mattson MP (2019). Effects of intermittent fasting on health, aging, and disease. N. Engl. J. Med..

[7] Gao Y, Tsintzas K, Macdonald IA, Cordon SM, Taylor MA (2022). Effects of intermittent (5:2) or continuous energy restriction on basal and postprandial metabolism: A randomised study in normal-weight, young participants. Eur. J. Clin. Nutr..

[8] Kanda T (2018). Apoptosis and non-alcoholic fatty liver diseases. World J. Gastroenterol..

[9] Jankauskas SS, Wong DWL, Bucala R, Djudjaj S, Boor P (2019). Evolving complexity of MIF signaling. Cell Signal..

[10] Heinrichs D (2011). Macrophage migration inhibitory factor (MIF) exerts antifibrotic effects in experimental liver fibrosis via CD74. Proc. Natl. Acad. Sci. U. S. A..

[11] Gligorovska L (2021). Macrophage migration inhibitory factor deficiency aggravates effects of fructose-enriched diet on lipid metabolism in the mouse liver. BioFactors.

[12] Qi D (2009). Cardiac macrophage migration inhibitory factor inhibits JNK pathway activation and injury during ischemia/reperfusion. J. Clin. Investig..

[13] Cui N (2022). Exercise inhibits JNK pathway activation and lipotoxicity via macrophage migration inhibitory factor in nonalcoholic fatty liver disease. Front. Endocrinol. (Lausanne)..

[14] Chao CH, Chen HR, Chuang YC, Yeh TM (2018). Macrophage migration inhibitory factor-induced autophagy contributes to thrombin-triggered endothelial hyperpermeability in sepsis. Shock.

[15] Li R (2021). The Role of macrophage migration inhibitory factor (MIF) in asthmatic airway remodeling. Allergy Asthma Immunol. Res..

[16] Gao Y (2020). Exercise and dietary intervention ameliorate high-fat diet-induced NAFLD and liver aging by inducing lipophagy. Redox Biol..

[17] Moon HY, Song P, Choi CS, Ryu SH, Suh PG (2013). Involvement of exercise-induced macrophage migration inhibitory factor in the prevention of fatty liver disease. J. Endocrinol..

[18] Ito M (2007). Longitudinal analysis of murine steatohepatitis model induced by chronic exposure to high-fat diet. Hepatol. Res..

[19] Lau JK, Zhang X, Yu J (2017). Animal models of non-alcoholic fatty liver disease: Current perspectives and recent advances. J. Pathol..

[20] Allaire M, Rautou PE, Codogno P, Lotersztajn S (2019). Autophagy in liver diseases: Time for translation?. J. Hepatol..

[21] Phillips JA (2021). Dietary guidelines for Americans, 2020–2025. Workplace Health Saf..

[22] Ok DP, Ko K, Bae JY (2018). Exercise without dietary changes alleviates nonalcoholic fatty liver disease without weight loss benefits. Lipids Health Dis..

[23] Różański G (2021). Effect of different types of intermittent fasting on biochemical and anthropometric parameters among patients with metabolic-associated fatty liver disease (MAFLD)—A systematic review. Nutrients.

[24] Stekovic S (2019). Alternate day fasting improves physiological and molecular markers of aging in healthy, Non-obese humans. Cell Metab..

[25] Johari MI (2020). Author Correction: A randomised controlled trial on the effectiveness and adherence of modified alternate-day calorie restriction in improving activity of non-alcoholic fatty liver disease. Sci. Rep..

[26] Ezpeleta M (2023). Effect of alternate day fasting combined with aerobic exercise on non-alcoholic fatty liver disease: A randomized controlled trial. Cell Metab..

[27] Zhang H (2020). Alternate-day fasting alleviates diabetes-induced glycolipid metabolism disorders: Roles of FGF21 and bile acids. J. Nutr. Biochem..

[28] Liu B, Page AJ, Hutchison AT, Wittert GA, Heilbronn LK (2019). Intermittent fasting increases energy expenditure and promotes adipose tissue browning in mice. Nutrition.

[29] Ma H (2010). Impaired macrophage migration inhibitory factor-AMP-activated protein kinase activation and ischemic recovery in the senescent heart. Circulation.

[30] Kleemann R (2000). Intracellular action of the cytokine MIF to modulate AP-1 activity and the cell cycle through Jab1. Nature.

[31] Miller EJ (2008). Macrophage migration inhibitory factor stimulates AMP-activated protein kinase in the ischaemic heart. Nature.

[32] Simons D (2011). Hypoxia-induced endothelial secretion of macrophage migration inhibitory factor and role in endothelial progenitor cell recruitment. J. Cell Mol. Med..

[33] Li H (2021). Exercise improves lipid droplet metabolism disorder through activation of AMPK-mediated lipophagy in NAFLD. Life Sci..

[34] Kleiner DE (2005). Design and validation of a histological scoring system for nonalcoholic fatty liver disease. Hepatology.

[35] You B (2020). Anti-insulin resistance effects of salidroside through mitochondrial quality control. J. Endocrinol..

[36] Matthews DR (1985). Homeostasis model assessment: Insulin resistance and beta-cell function from fasting plasma glucose and insulin concentrations in man. Diabetologia.

