# Supplementary material for: Intermittent fasting activates macrophage migration inhibitory factor and alleviates high-fat diet-induced nonalcoholic fatty liver disease
Source: Sci Rep. 2023 Aug 11;13:13068. doi: 10.1038/s41598-023-40373-5 (PMC10421944; doi:10.1038/s41598-023-40373-5)
Supplement: Supplementary file 1 — Supplementary Information. [file 41598_2023_40373_MOESM1_ESM.pdf]

DATA IN BRIEF

Data of Figure 1

Food intake(g/2d/mouse)	ND	HFD	HFD+ND	HFD+IF
	10.89	11.45	8.18	8.05
	9.82	12.27	10.91	9.00
	10.91	10.45	9.73	8.91
	10.91	9.82	13.35	8.73

liverweight (g)	ND	HFD	HFD+ND	HFD+IF
	1.13	1.29	1.12	1.17
	1.10	1.09	1.28	1.01
	1.00	1.07	1.20	0.83
	0.97	1.35	1.25	1.00
	1.02	1.42	1.07	1.01
	0.89	1.20	1.04	0.96

bodyweight (g)	ND	HFD	HFD+ND	HFD+IF
8w	19.71	21.77	22.68	20.95
	20.17	21.43	22.52	21.85
	25.89	22.07	19.88	21.38
	19.58	21.84	22.53	22.61
	22.39	22.58	21.41	21.62
	21.62	22.07	22.33	21.76
19w	28.40	29.70	30.50	31.50
	27.20	29.30	29.30	30.10
	26.80	31.90	32.50	30.40
	28.60	33.50	32.80	30.10
	27.90	31.30	28.70	29.20
	28.00	31.60	27.10	29.60
31w	31.00	38.00	31.90	31.80
	32.90	34.20	28.00	27.60
	28.90	43.10	30.10	31.90
	31.10	34.30	28.70	30.80
	31.80	34.30	29.90	32.00
	30.10	39.60	31.10	30.40

Red oil O area (%)	ND	HFD	HFD+ND	HFD+IF

	7.58	23.55	17.86	13.77
	5.37	27.03	14.80	10.85
	6.33	26.69	16.31	9.63

Data of Figure 2

	ND	HFD	HFD-ND	HFD- IF
FBS (mmol/L)	4.30	9.30	7.00	6.80
	6.70	7.90	7.90	5.60
	5.50	9.30	8.40	7.30
	5.50	10.30	8.10	4.80
	6.80	9.40	7.70	6.80
	7.10	9.80	9.10	5.90

	ND	HFD	HFD-ND	HFD- IF
FINS (mU/L)	22.39	28.85	27.74	23.65
	17.82	35.12	28.78	25.43
	25.68	32.74	29.18	16.66
	22.27	28.30	26.02	19.05
	20.20	31.35	26.48	20.89
	18.61	27.17	27.63	21.92

	ND	HFD	HFD-ND	HFD- IF
HOMA-IR index	4.28	11.92	8.63	7.15
	5.31	12.33	10.11	6.33
	6.28	13.53	10.90	5.40
	5.44	12.95	9.37	4.06
	6.10	13.10	9.06	6.31
	5.87	13.66	11.17	5.75

protein expression	ND	HFD	HFD-ND	HFD- IF
P-IRS/IRS	1.10	0.26	0.50	0.66
	1.00	0.17	0.37	0.56
	0.90	0.22	0.34	0.52
P-AKT/AKT	1.09	0.16	0.42	0.72
	0.89	0.20	0.39	0.64
	1.01	0.14	0.25	0.58
	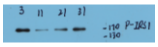	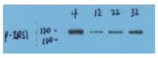	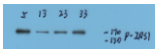	
	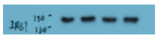	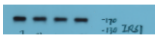	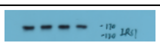	
	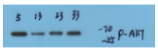	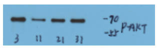	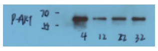	

	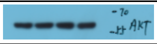	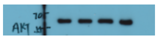	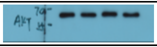	

mRNA expression	CON	HF	HFCON	IF
SREBP1C	0.13	0.91	0.72	0.45
	0.18	1.03	0.77	0.31
	0.12	1.07	0.64	0.33
FAS	0.16	0.99	0.51	0.50
	0.23	0.98	0.62	0.42
	0.15	1.03	0.71	0.47
		SREBP1C	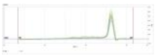	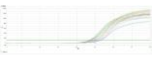
		FAS	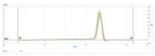	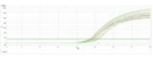
		GAPDH	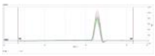	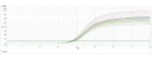

mRNA expression	CON	HF	HFCON	IF
CPT1	1.00	0.21	0.52	0.78
	0.84	0.16	0.57	0.50
	1.16	0.14	0.48	0.60
CD36	0.24	1.09	0.64	0.60
	0.17	0.96	0.68	0.41
	0.14	0.95	0.73	0.46
ACC	0.13	1.06	0.40	0.33
	0.09	0.82	0.57	0.25
	0.08	1.12	0.54	0.37
		CPT1	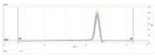	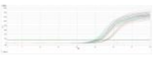
		CD36	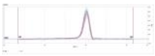	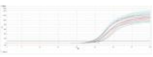
		ACC	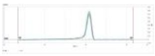	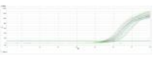
		GAPDH	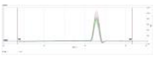	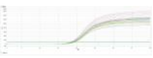

Data of Figure 3

tunel+ cell (%)	ND	HFD	HFD-ND	HFD-IF
	11.86	55.79	34.01	23.75
	6.59	62.44	47.15	12.26
	9.66	42.92	31.25	13.95

protein expression	ND	HFD	HFD-ND	HFD-IF

BAD	0.22	0.89	0.71	0.32
	0.32	1.06	0.67	0.48
	0.30	1.04	0.80	0.47
BAX	0.25	0.95	0.56	0.41
	0.25	1.02	0.61	0.38
	0.18	1.04	0.65	0.47
Bcl-2	1.04	0.20	0.33	0.63
	1.00	0.20	0.43	0.49
	0.96	0.12	0.47	0.57
	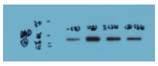	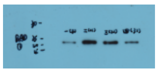	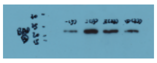	
	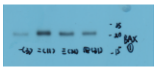	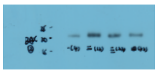	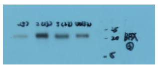	
	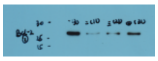	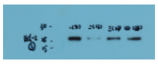	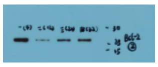	
	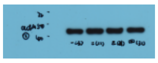	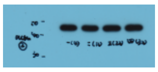	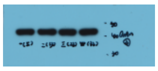	

Data of Figure 4

protein expression	ND	HFD	HFD-ND	HFD-IF
MIF	1.10	0.19	0.38	0.61
	0.99	0.18	0.35	0.57
	0.92	0.22	0.36	0.57
CD74	1.00	0.20	0.39	0.59
	1.04	0.20	0.41	0.57
	1.08	0.24	0.49	0.61
	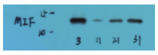	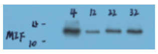	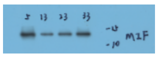	
	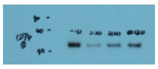	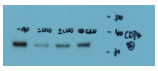	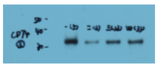	
	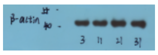	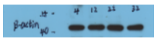	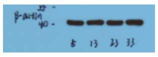	

protein expression	ND	HFD	HFD-ND	HFD-IF
p-MKK4/MKK4	0.40	0.99	0.84	0.53
	0.29	0.98	0.84	0.59
	0.32	1.03	0.78	0.52
p-JNK54/JNK	0.17	0.99	0.81	0.20
	0.26	0.91	0.62	0.35
	0.14	1.10	0.79	0.52
p-JNK46/JNK	0.23	0.92	0.77	0.27

	0.27	0.88	0.68	0.36
	0.13	1.20	0.79	0.49
	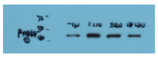	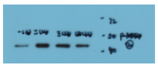	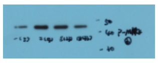	
	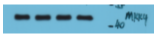	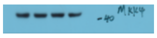	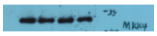	
	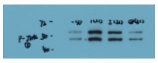	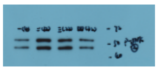	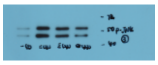	
	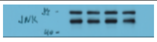	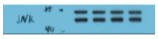	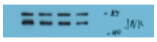	

Data of Figure 5

protein expression	ND	HFD	HFD-ND	HFD-IF
p-AMPK/AMPK	1.02	0.19	0.42	0.68
	0.85	0.21	0.41	0.61
	1.14	0.26	0.49	0.78
SIRT1	1.03	0.28	0.49	0.75
	1.03	0.24	0.47	0.85
	0.95	0.22	0.39	0.71
	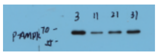	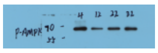	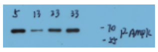	
	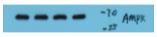	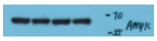	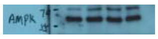	
	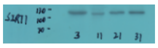	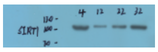	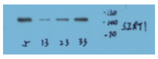	
	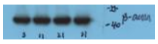	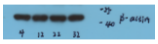	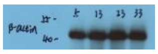	

protein expression	ND	HFD	HFD-ND	HFD-IF
P62	0.39	1.05	0.75	0.58
	0.25	0.97	0.77	0.53
	0.22	0.97	0.68	0.39
p-ULK1/ULK	0.99	0.12	0.53	0.93
	0.71	0.08	0.28	0.53
	1.30	0.12	0.29	0.53
LC3II/I	0.85	0.14	0.31	0.52
	1.03	0.12	0.31	0.53
	1.11	0.14	0.32	0.48
	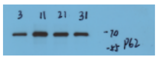	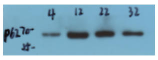	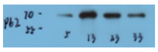	
	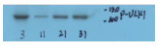	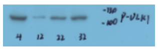	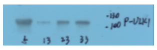	
	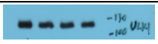	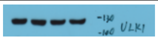	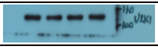	

	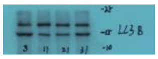LC3B	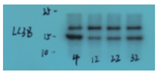LC3B	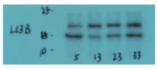LC3B	
	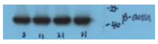	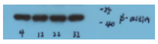	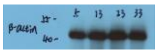	
